# Cembranoids from Octocoral *Lobophytum crassum* (von Marenzeller, 1886)

**DOI:** 10.3390/md19030130

**Published:** 2021-02-27

**Authors:** Yao-Tsung Yeh, Sung-Chun Lin, Gene-Hsiang Lee, Zhi-Hong Wen, Tsong-Long Hwang, Yu-Jen Wu, Jih-Jung Chen, Lee-Shing Fang, Mei-Kang Yuan, Ping-Jyun Sung

**Affiliations:** 1 Graduate Institute of Marine Biology, National Dong Hwa University, Pingtung 944401, Taiwan; 610963011@gms.ndhu.edu.tw; 2 National Museum of Marine Biology and Aquarium, Pingtung 944401, Taiwan; 3 Department of Orthopaedic Surgery, Ping-Tung Christian Hospital, Pingtung 900026, Taiwan; 2572@ptch.org.tw; 4 Instrumentation Center, National Taiwan University, Taipei 106319, Taiwan; ghlee@ntu.edu.tw; 5 Department of Marine Biotechnology and Resources, National Sun Yat-sen University, Kaohsiung 804201, Taiwan; wzh@mail.nsysu.edu.tw (Z.-H.W.); lsfang@gcloud.csu.edu.tw (L.-S.F.); 6 Institute of BioPharmaceutical Sciences, National Sun Yat-sen University, Kaohsiung 804201, Taiwan; 7 Graduate Institute of Natural Products, College of Medicine, Chang Gung University, Taoyuan 333323, Taiwan; htl@mail.cgu.edu.tw; 8 Research Center for Chinese Herbal Medicine, Graduate Institute of Healthy Industry Technology, College of Human Ecology, Chang Gung University of Science and Technology, Taoyuan 333324, Taiwan; 9 Department of Anaesthesiology, Chang Gung Memorial Hospital, Taoyuan 333423, Taiwan; 10 Department of Chemical Engineering, Ming Chi University of Technology, New Taipei City 243303, Taiwan; 11 Department of Food Science and Nutrition, Meiho University, Pingtung 912009, Taiwan; x00002180@meiho.edu.tw; 12 Faculty of Pharmacy, School of Pharmaceutical Sciences, National Yang Ming Chiao Tung University, Taipei 112304, Taiwan; jjungchen@nycu.edu.tw; 13 Center for Environmental Toxin and Emerging-Contaminant Research, Cheng Shiu University, Kaohsiung 833301, Taiwan; 14 Super Micro Mass Research and Technology Center, Cheng Shiu University, Kaohsiung 833301, Taiwan; 15 Department of Medical Imaging and Radiology, Shu-Zen Junior College of Medicine and Management, Kaohsiung 821004, Taiwan; 16 Department of Radiology, An Nan Hospital, China Medical University, Tainan 709204, Taiwan; 17 Chinese Medicine Research and Development Center, China Medical University Hospital, Taichung 404394, Taiwan; 18 Graduate Institute of Natural Products, Kaohsiung Medical University, Kaohsiung 807378, Taiwan

**Keywords:** *Lobophytum crassum*, cembranoid, lobocrassin, lobohedleolide, X-ray, anti-inflammation, iNOS

## Abstract

Two cembranoids, including a new compound, lobocrassin I (**1**), as well as a known analogue, lobohedleolide (**2**), were obtained by solvent extraction from octocoral *Lobophytum crassum*. This study employed a spectroscopic approach to establish the structures of these two cembranoids, and utilized single-crystal X-ray diffraction analysis to determine their absolute configurations. The results of biological activity assays demonstrated that cembranoid **2** exhibited bioactivity against the protein expressions of inducible nitric oxide synthase (iNOS) lipopolysaccharide (LPS)-treated RAW 264.7 mouse macrophage cells.

## 1. Introduction

**Figure 1:**
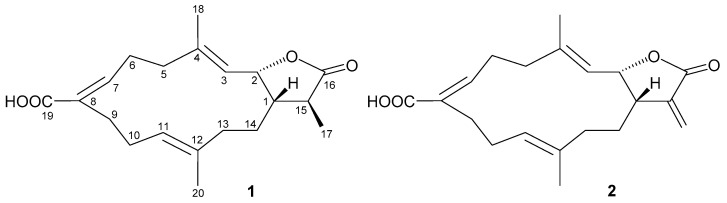
Structures of lobocrassin I (**1**) and lobohedleolide (**2**).

Cembrane-type diterpenoids are a group of 14-membered macrolides obtained from terrestrial and marine organisms, with novel structures and extensive bioactivities [1]. Octocorals belonging to the genera Lobophytum, Sarcophyton, and Sinularia are currently known to be critical sources for the supply of cembranoids [2]. In connection with our continuing studies of marine invertebrates with biomedical potential, we have focused considerable attention on invertebrates found in the marine habitat of the waters around Taiwan, with the aim of informing new drug development. In this research, we completed the preparation, structural identification, and anti-inflammatory activity assessment of a new cembranoid, lobocrassin I (**1**), as well as a known cembranoid, lobohedleolide (**2**) [3,4,5,6] (Figure 1), obtained from *L. crassum* (von Marenzeller, 1886) [7,8]. *Lobophytum crassum* is a rich cembranoid-containing octocoral distributed extensively in tropical Indo-Pacific Ocean, including Taiwanese waters where the Kuroshio current and South China Sea surface current converge to provide high biodiversity and the cembrane-type diterpenoids prepared from soft coral *L. crassum* were proven to have the potential to be used as therapeutic agents to treat inflammation [9,10,11,12,13,14,15]. This paper reported details of the isolation, structure determination, and biological evaluation of cembranoids **1** (lobocrassin I) and **2** (lobohedleolide) (Figure 1). 

## 2. Results and Discussion

Freshly-collected *L. crassum* was frozen and subsequently freeze-dried, powdered, and extracted with a solvent mixture of methanol/dichloromethane (MeOH/CH_2_Cl_2_) at a 1:1 ratio to give an extract that was subsequently separated by organic solvent ethyl acetate (EtOAc)-water partitioning. The EtOAc layer was collected and loaded onto a column chromatograph with silica gel, and subsequently separated using high performance liquid chromatography (HPLC), yielding cembranoids **1** and **2**. The known compound was elucidated as lobohedleolide (**2**) by analysis of its spectroscopic data and comparison with previously reported values [3].

Lobocrassin I (**1**) was obtained as colorless prisms. The positive mode high resolution electrospray ionization mass spectrum ((+)-HRESIMS) showed a peak at *m/z* 355.18815, suggesting the molecular formula C_20_H_28_O_4_ (calcd. for C_20_H_28_O_4_ + Na, 355.18798), indicating seven degrees of unsaturation in the compound. The IR spectrum revealed absorptions for α,β-unsaturated carboxyl (ν_max_ 3749~2216 and 1710 cm^–1^) and γ- lactone (ν_max_ 1766 cm^–1^) groups. The ^13^C NMR spectrum of **1** (Table 1) showed signals of 20 carbons. The multiplicities of carbon signals were determined from a distortionless enhancement by polarization transfer (DEPT)/heteronuclear single quantum coherence (HSQC) spectrum, indicating three methyls, six sp^3^ methylenes, three sp^3^ methines (one bearing a heteroatom), three sp^2^ methines, and five sp^2^ non-protonated carbons (two carbonyls and three olefins). From the ^1^H and ^13^C NMR spectra (Table 1), **1** was found to possess a γ-lactone (δ_C_ 179.3, C-16), a carboxyl carbon (δ_C_ 170.7, C-19), and three tri- substituted olefins (δ_H_ 5.74, 1H, dd, *J* = 8.4, 3.6 Hz, H-7; 5.21, 1H, d, *J* = 10.8 Hz, H-3; 4.93, 1H, dd, *J* = 8.8, 7.6 Hz, H-11; δ_C_ 119.6, CH-3; 142.4, C-4; 147.8, CH-7; 128.7, C-8; 122.7, CH-11; 135.5, C-12). Five double bonds that accounted for the five degrees of unsaturation were identified. The remaining two degrees of unsaturation delineated the configuration, and indicated that **1** was a bicyclic molecule.

**Figure 2:**
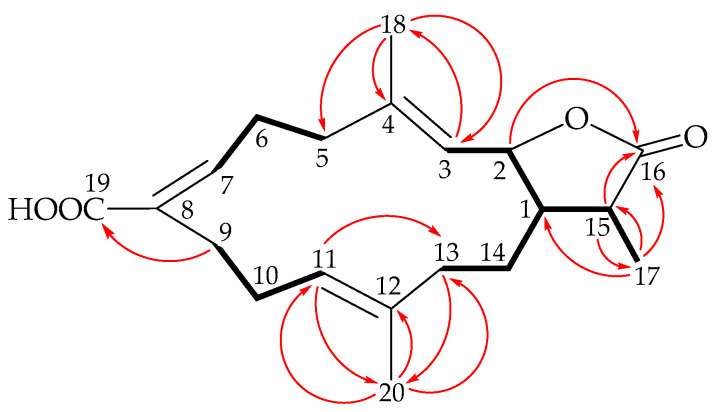
Major COSY (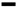) and HMBC (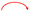) correlations of **1**.

The H_2_-13/H_2_-14/H-1/H-2/H-3, H_2_-5/H_2_-6/H-7, H_2_-9/H_2_-10/H-11, and H-1/H-15/H_3_-17 spin systems identified by ^1^H-^1^H correlation spectroscopy (COSY) (Figure 2) were fit to the regiochemistry of vicinal couplings in **1**. Based on the aforementioned data and the results of ^1^H-^13^C long-range correlations obtained from heteronuclear multiple-bond coherence (HMBC) analysis, the molecular framework of **1** was determined (Figure 2). The vinyl methyls at C-4 and C-12 were established from HMBC correlations between H_3_-18/C-3, C-4, C-5, and H_3_-20/C-11, C-12, C-13, respectively. The carboxyl group at C-8 was established from an HMBC correlation between one of the C-9 methylene protons (δ_H_ 1.84) and the carbonyl carbon of carboxyl group at δ_C_ 170.7 (C-19). An ester carbonyl signal at δ_C_ 179.3 (C-16) showed a ^2^*J*-coupling with a methine proton at δ_H_ 2.33 (H-15); ^3^*J*- couplings with an oxymethine proton at δ_H_ 5.32 (H-2), and methyl protons at δ_H_ 1.25 (H_3_-17) in the HMBC spectrum established the α-methyl-γ-lactone moiety in **1**.

**Figure 3:**
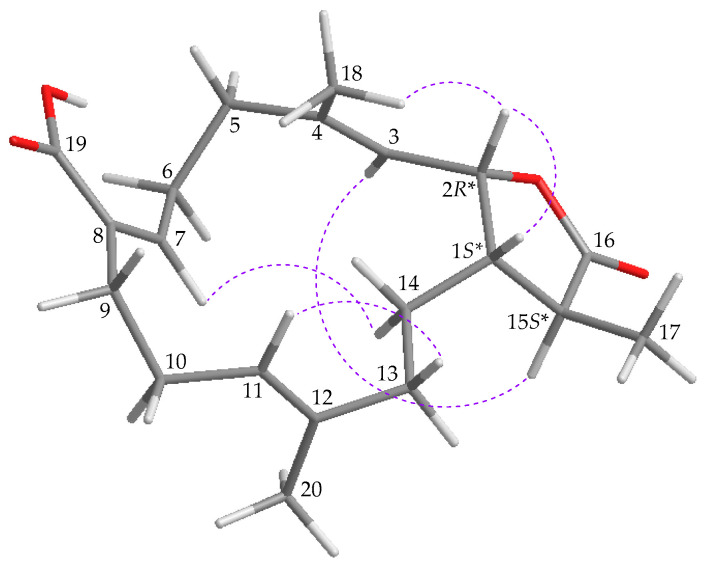
Major protons with NOESY (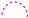) correlations of **1**.

The interactions obtained using nuclear Overhauser effect spectroscopy (NOESY) and the data of vicinal ^1^H-^1^H coupling constants revealed the relative stereochemistry of **1** (Figure 3). Biogenetically, in most cases the proton at C-1 is β-oriented in naturally-occurring cembranoids from *Lobophytum* spp. [2]. The NOESY cross-peak of H-1 and H-2 suggested that these two protons were β-oriented. The vinyl methyl H_3_-18 exhibited a NOESY response with H-2, but not with H-3, and a NOESY correlation was observed between H-3 and H-15, demonstrating the *trans* configuration of ∆^3^, and H-15 was therefore α-oriented in the γ-lactone moiety. The *trans* relationship between H-1 and H-15 was established from a large coupling constant (*J* = 12.0 Hz) for these two vicinal protons. H-11 showed a NOESY correlation with one of the C-13 methylene protons (δ_H_ 2.01), but not with H_3_-20, indicating the *trans* configuration of ∆^11^. Furthermore, olefin proton H-7 exhibited a NOESY correlation with one of the C-14 methylene protons (δ_H_ 1.82), also demonstrating the *cis* geometry of ∆^7^. Based on the aforementioned results, the configurations of the stereogenic centers of **1** were assigned as (1S*,2R*,15S*) (Supplementary Materials, Figures S1–S10).

**Figure 4:**
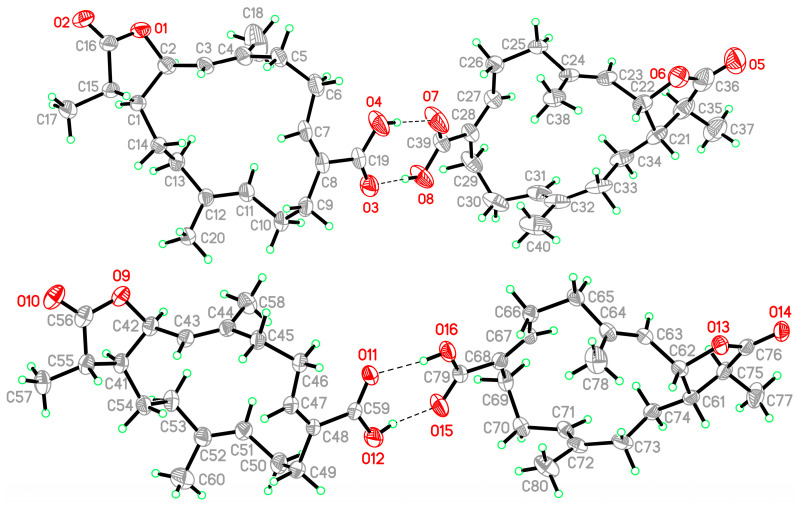
ORTEP plot revealing the absolute configuration of **1**.

Single-crystal X-ray diffraction was used to confirm the structure of **1**. The data suggested *E*-geometries of the C-3/4, C-11/12, and *Z*-geometry of the C-7/8 carbon-carbon double bonds in **1**; in addition, the absolute configurations of the stereogenic carbons of **1** were confirmed as (1*S*,2*R*,15*S*) based on an Oak Ridge thermal-ellipsoid plot (ORTEP) of **1** (Figure 4). According to the X-ray determined structure of **1**, the carboxylic acids formed dimers, in which the monomer units were held together by hydrogen bonds.

Cembranoid **2** was obtained as colorless prisms, showing a sodiated ESIMS quasimolecular ion peak at *m/z* 353, and was found to have the molecular formula C_20_H_26_O_4_ by analysis of ^13^C and ^1^H NMR data (see Materials and Methods). The result revealed that this compound had 8 degrees of unsaturation. Strong bands at 3665~2398 (broad), 1760, and 1682 cm^−1^ in the IR spectrum indicated the presence of α,β-unsaturated carboxyl and γ-lactone groups. The ^13^C NMR and DEPT spectra revealed that **2** had 20 carbons, including two methyls, six sp^3^ methylenes, one sp^2^ methylene, two sp^3^ methines (one bearing a heteroatom), three sp^2^ methines, and six sp^2^ non-protonated carbons (one ester carbonyl and one carboxyl group). Therefore, **2** was identified as having two rings. It was found that the NMR data of **2** were almost identical to those of a known compound, lobohedleolide [3], and these two compounds were found to possess positive optical rotation value, suggesting that compound **2** is lobohedleolide, although the ^13^C chemical shift for the carboxyl carbon in **2** (δ_C_ 171.7, C-19) was different from that reported (δ_C_ 173.2) [3] (Supplementary Materials, Figures S11–S19). 

**Figure 5:**
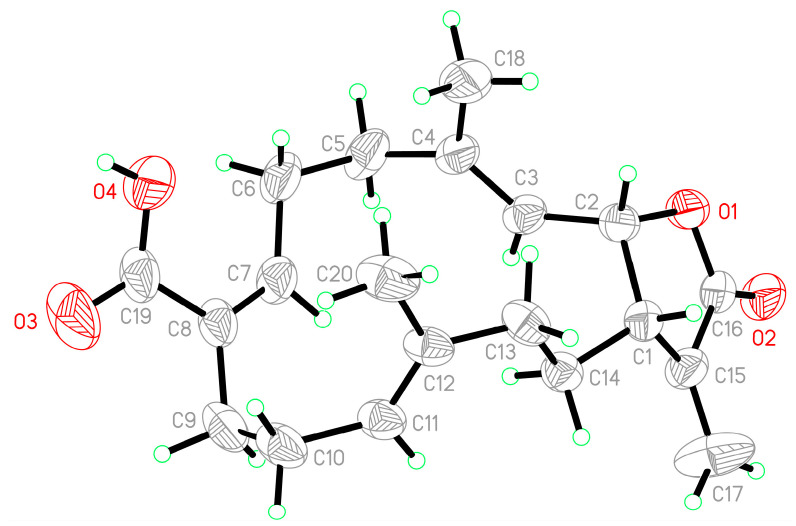
ORTEP plot revealing the absolute configuration of **2**.

Lobohedleolide (**2**) was first isolated from octocoral *Lobophytum hedleyi*, collected from the coral reefs of Yayeyama Islands of Okinawa, Japan [3]. The structure of lobohedleolide was revealed by spectroscopic analysis, and its absolute configuration was concluded from X-ray study of its *p*-bromophenacyl ester derivative [3]. In this study, the structure of **2** was determined by single-crystal X-ray diffraction directly for the first time, and the absolute configuration for this compound was elucidated as (1*S*,2*S*) (Figure 5). Because the structure of **2** has been established by a single-crystal X-ray diffraction analysis, the authors suggested that the ^13^C chemical shift of carboxyl carbon in structure for lobohedleolide should be re-examined [3].

The anti-inflammatory effects of lobocrassin I (**1**) and lobohedleolide (**2**) were assessed by measuring their effects on proinflammatory proteins/enzymes inducible nitric oxide synthase (iNOS) and cyclooxygenase 2 (COX-2) production from lipopoly-saccharides (LPS)-stimulated RAW264.7 cells (a murine macrophage cell line). Western blotting results showed that cembranoid **2** at 10 µM inhibited iNOS expression, which reduced the protein level to 28.50%, as compared with cells in the control group treated with LPS only (Table 2). In previous studies, cembranoid **2** was reported to exhibit extensive bioactivities such as cytotoxic [3,5], antiviral [4,16,17], and anti-inflammatory [16,18,19,20] activities, and the anti-inflammatory activity of **2** has been evaluated and revealed that **2** is effective against both carrageenin induced edema and cotton pellet implantation rat models [18]. Lobocrassin I (**1**) did not show activity, implying that the exo-methylene substituent at C-15 enhanced the bioactivity in comparison with cembranoid **2**.

## 3. Materials and Methods

### 3.1. General Experimental Procedures

A digital polarimeter (model P-1010; JASCO Corp., Tokyo, Japan) was used to determine optical rotations of the samples. IR spectra were collected using a spectro-photometer (model Nicolet iS5 FT-IR; Thermo Fisher Scientific, Waltham, MA, USA). ^1^H and ^13^C NMR spectra were recorded on an ECZ-400 spectrometer (Jeol Ltd., Tokyo, Japan) for solutions in CDCl_3_ (with residual CHCl_3_ (δ_H_ 7.26 ppm) and CDCl_3_ (δ_C_ 77.0 ppm) as internal standards). For coupling constants (*J*), the results were given in frequency units, Hz. For ESIMS and HRESIMS, the results were obtained using a SolariX FTMS mass spectrometer (7 Tesla; Bruker, Bremen, Germany). The extracted samples were separated by column chromatography with silica gel (range, 230 to 400 mesh; Merck, Darmstadt, Germany). The purity of a compound can be determined by Thin-layer chromatography (TLC), a method for analyzing mixtures by separating the compounds in the mixture. The TLC plates with silica gel coated with fluorescent indicator F_254_ were employed. For visualization, the plates were charred with 10% (*v/v*) aqueous sulfuric acid solution, then heated at 105 °C until spots were seen. For normal-phase HPLC separation, a system containing a pump (Hitachi model L-7110; Tokyo, Japan) and an injection interface (No. 7725; Rheodyne) was employed, which was equipped with a semi-prep column with a dimension of 2 × 25 cm, 5 μm particle size (Sigma, St. Louis, MO, USA). For reverse-phase HPLC separation, a system composed of a pump (Hitachi model L-2130, Tokyo, Japan) and a diode-array detector (LaChrom L-2455, Hitachi, Tokyo, Japan) were used, which was equipped with a column with a dimension of 2.1 × 25 cm, 5 μm particle size (Phenomenex, Torrance, CA, USA).

### 3.2. Soft Coral Specimens

The soft coral *L. crassum* was manually collected by an underwater diver with a breathing apparatus from the marine habitat around Southern Taiwan on 23 July 2020. The specimens were frozen directly after harvesting. Identification of the specimens was performed by one of the authors of this study (Y.-T.Y.) by assessment of the features and comparison with the characteristics reported in the literature [7,8]. A representative sample of the soft coral (voucher no.: NMMBA-TW-SC-2020-0723) was stored in the National Museum of Marine Biology and Aquarium, Taiwan.

### 3.3. Cembranoid Compound Preparation

*Lobophytum crassum* (wet/dry weight = 1174 g/591 g) was crushed and then extracted with a mixture of MeOH and CH_2_Cl_2_ (1:1) to give an extract (22.4 g), that was next separated by solvent-partition with EtOAc and H_2_O. The EtOAc extract (7.34 g) was then placed in an SiO_2_ column and washed with an eluent of hexanes/EtOAc (by stepwise-gradient increase from 100:1—pure EtOAc) to yield 12 fractions A−L. Fraction J (294 mg) was then purified by semi-prep normal-phase HPLC to give eight fractions J1–J8. Fraction J3 (161.0 mg) was then purified by semi-prep reverse-phase HPLC (MeOH:H_2_O = 65:35 (*v/v*); at a rate of 5.0 mL/min) to afford compounds **2** (47.1 mg) and **1** (16.6 mg).

Lobocrassin I (**1**): Colorless prisms; ${\left[\alpha \right]}_{D}^{22}$ + 46 (*c* 0.03, CHCl_3_); IR (ATR) ν_max_ 3749~2216 (broad), 1766, 1710 cm^−1^; ^1^H NMR (400 MHz, CDCl_3_) and ^13^C NMR (100 MHz, CDCl_3_) data, see Table 1; ESIMS: *m/z* 355 [M + Na]^+^; HRESIMS: *m/z* 355.18815 (calcd. for C_20_H_28_O_4_ + Na, 355.18798).

Lobohedleolide (**2**): Colorless prisms; ${\left[\alpha \right]}_{D}^{22}$ + 48 (*c* 0.21, CHCl_3_) (ref. [3] ${\left[\alpha \right]}_{D}$ + 104.2 (*c* 1.12, CHCl_3_); ref. [4] ${\left[\alpha \right]}_{D}$ + 97.3 (*c* 0.38, CHCl_3_)); IR (ATR) ν_max_ 3665~2398 (broad), 1760, 1682 cm^−1^; ^1^H NMR (400 MHz, CDCl_3_) and ^13^C NMR (100 MHz, CDCl_3_) data, see Table 1; ESIMS: *m/z* 353 [M + Na]^+^.

### 3.4. Single-Crystal X-ray Crystallography of Lobocrassin I (**1**)

Suitable colorless prisms of **1** were acquired from EtOA/acetone (4:1). The crystal (0.256 × 0.210 × 0.210 mm^3^) belongs to the monoclinic system, space group *P*2_1_ (#4), with *a* = 19.0021(4) Å, *b* = 9.3796(2) Å, *c* = 22.1329(5) Å, *V* = 3810.26(14) Å^3^, *Z* = 8, *D*_calcd_ = 1.159 Mg/m^3^, and *λ* (Mo Kα) = 0.71073 Å. Intensity data (up to *θ*_max_ of 27.5°) were obtained using an X-ray diffractometer (Bruker D8 Venture, Bremen, Germany). All 30,010 reflections were collected. By using direct methods and a full-matrix least-squares refinement [21,22], the structure of compound **1** was obtained. The refined structural model converged to a final R1 = 0.0546 and wR2 = 0.1277 for 17,444 observed reflections, with *I* > 2σ(*I*) and 881 variable parameters. The absolute configuration of this molecule was confirmed by an absolute structure Flack parameter, x = 0.0(4) [23,24]. Crystallographic data of compound **1** have been deposited with the Cambridge Crystallographic Data Center (CCDC) as supplementary publication no. CCDC 2053256 [25].

### 3.5. Single-Crystal X-ray Crystallography of Lobohedleolide (**2**)

Suitable colorless prisms of **2** were acquired from MeOH. The crystal (0.324 × 0.193 × 0.152 mm^3^) belongs to the orthorhombic system, space group *P*2_1_2_1_2_1_ (#19), with *a* = 8.8283(4) Å, *b* = 12.4788(6) Å, *c* = 16.8460(7) Å, *V* = 1855.87(14) Å^3^, *Z* = 4, *D*_calcd_ = 1.183 Mg/m^3^, and *λ* (Mo Kα) = 0.71073 Å. Intensity data (up to *θ*_max_ of 30.0°) were obtained using an X-ray diffractometer (Bruker D8 Venture, Bremen, Germany). All 12,446 reflections were collected. The structure of compound **2** was obtained using the same processes as described above for compound **1**. The refined structural model converged to a final R1 = 0.0502 and wR2 = 0.1294 for 5399 observed reflections, with *I* > 2σ(*I*) and 220 variable parameters. The absolute configuration of this molecule was confirmed by Flack parameter x = −0.1(5) [23,24]. Crystallographic data for the structure of lobohedleolide (**2**) were also deposited with the CCDC as supplementary publication no. CCDC 2043446 [25].

### 3.6. In Vitro Anti-inflammatory Assay

Murine RAW 264.7 macrophages were obtained from the American Type Culture Collection (ATCC; No. TIB-71). Inflammation in macrophages was induced by incubating them for 16 h in a medium containing only LPS (0.01 µg/mL) without compounds. For the anti-inflammatory activity assay, compounds (10 µM) were added to the cells 5 min before LPS challenge. The cells were then washed with ice cold phosphate-buffered saline (PBS), lysed in ice-cold lysis buffer (50 mM Tris, pH 7.5, 150 mM NaCl, 1% Triton X-100, 100 µg/mL phenylmethylsulfonyl fluoride, 1 µg/mL aprotinin), and then centrifuged at 20,000× *g* for 30 min at 4 °C. The supernatant was decanted from the pellet and retained for Western blot analysis of pro-inflammation inducible nitric oxide synthase (iNOS) and cyclooxygenase-2 (COX-2) protein expression. Protein concentrations were determined using the detergent compatible (DC) protein assay kit (Bio-Rad, Hercules, CA, USA). Western blotting was performed according to the method described in a previous study [26]. An equal volume of sample buffer (2% 2-mercaptoethanol, 2% sodium dodecyl sulfate (SDS), 0.1% bromophenol blue, 10% glycerol, and 50 mM Tris-HCl (pH 7.2)) was added to the samples, and the protein lysates were loaded onto a 10% SDS- polyacrylamide gel. Electrophoresis was carried out at 150 V for 90 min. After electro-phoresis, gels were transferred overnight at 4 °C in transfer buffer (380 mM glycine, 50 mM Tris-HCl, 1% SDS and 20% methanol) onto a polyvinylidene difluoride membrane (PVDF; Immobilon-P, Millipore Corp., Billerica, MA, USA (0.45 µm pore size)). The PVDF membrane was first blocked with 5% non-fat dry milk in Tris-buffered saline containing 0.1% Tween (TTBS; 20 mM Tris-HCl, 0.1% Tween 20, and 137 mM NaCl (pH 7.4)) and incubated overnight at 4 °C with the primary antibodies for iNOS, COX-2, and β-actin proteins. Anti-iNOS and anti-COX-2 antibodies were purchased from Cayman Chemical Company (Ann Arbor, MI, USA). A horseradish peroxidase-conjugated secondary antibody was used for detection. It was obtained from Jackson ImmunoResearch Laboratories (West Grove, PA, USA). The bound antibodies were detected by chemiluminescence (Millipore Corp.). The images were obtained using the UVP BioChemi Imaging System, and the LabWorks 4.0 software (UVP, Upland, CA, USA) was used to quantify the relative densities.

## 4. Conclusions

A series of cembranoids, including lobocrassins A−F [27,28,29], have been obtained from this target organism. The structures of a new compound lobocrassin I (**1**) and its analogue lobohedleolide (**2**), two cembranoids isolated from *L. crassum* containing α,β-unsaturated carboxyl group and γ-lactone systems, were elucidated using spectroscopic methods and X-ray analysis. Cembranoid **2** exhibited effective inhibition of iNOS production. Structure-activity relationship (SAR) comparison also indicated that the exomethylene group at C-15 has an important role in the activity against the inflammatory response. We have started to optimize the aquaculture conditions for this octocoral in tanks, with the aim of culturing a large quantity of coral that is able to provide a stable supply of bioactive materials. This approach will protect the coral population in natural marine habitats from over-exploitation [30,31], and provide raw materials of consistent quality. 

## Figures and Tables

**Table 1:** ^1^H and ^13^C NMR data for cembranoids **1** and **2**.

	1		2	
Position	δ_H_^a^ (*J* in Hz)	δ_C_^b^	δ_H_^a^ (*J* in Hz)	δ_C_^b^
1	2.21 m	46.6, CH ^c^	3.11 m	42.9, CH ^c^
2	5.32 dd (10.8, 7.2)	77.6, CH	5.43 dd (10.4, 8.0)	77.9, CH
3	5.21 d (10.8)	119.6, CH	5.05 d (10.4)	120.5, CH
4		142.4, C		142.0, C
5/5’	2.38 m; 2.24 m	39.9, CH_2_	2.38 br d (14.0); 2.19 m	39.8, CH_2_
6/6’	3.11 m; 2.42 m	26.6, CH_2_	3.07 m; 2.46 br d (12.4)	26.6, CH_2_
7	5.74 dd (8.4, 3.6)	147.8, CH	5.72 dd (8.8, 4.0)	148.1, CH
8		128.7, C		128.8, C
9	2.75 br d (13.2); 1.82 m	35.2, CH_2_	2.71 br d (13.2); 1.87 ddd (13.2, 7.6, 7.6)	35.1, CH_2_
10	2.19 m	25.0, CH_2_	2.18 m	25.0, CH_2_
11	4.93 dd (8.8, 7.6)	122.7, CH	4.93 dd (8.4, 8.0)	122.8, CH
12		135.5, C		135.3, C
13/13’	2.01 m; 1.67 m	36.6, CH_2_	2.07 m; 1.69 m	36.1, CH_2_
14/14’	1.84 m; 1.35 m	27.8, CH_2_	1.99 ddd (12.8, 6.4, 3.2); 1.45 m	27.0, CH_2_
15	2.33 dq (12.0, 6.8)	38.8, CH		138.7, C
16		179.3, C		170.6, C
17a/b	1.25 d (6.8)	13.6, CH_3_	6.28 d (3.2); 5.54 d (3.2)	120.8, CH_2_
18	1.73 s	15.0, CH_3_	1.73 s	15.3, CH_3_
19		170.7, C		171.7, C
20	1.52 s	16.0, CH_3_	1.54 s	16.1, CH_3_

^a 1^H NMR (400 MHz, CDCl_3_), ^b 13^C NMR (100 MHz, CDCl_3_), ^c^ Multiplicity-edited ^13^C, DEPT, and HSQC spectra.

**Table 2:** Suppression effects of cembranoids **1** and **2** on iNOS and COX-2 protein/enzyme expressions in LPS-induced macrophages.

Compound/Treatment	iNOS		COX-2		*β*-Actin	
(10 µM)	Production Level					
Control	2.23	±0.87	1.02	±0.14	106.12	±4.17
Vehicle	100.01	±4.27	100.00	±2.62	100.00	±0.74
**1**	90.82	±2.16	110.85	±2.10	102.38	±2.12
**2**	28.50	±2.69	78.99	±3.36	100.45	±2.06
Dexamethasone	54.53	±3.58	17.66	±1.75	103.14	±2.46

Values of cells treated with LPS alone were set to 100% as the reference for normalization. Dexamethasone at 10 µM was used as a positive reference to treat cells. Experimental results are shown as the mean ± S.E.M. The β-actin of Western blotting is used for loading/internal control.

## Supplementary Materials

Supplementary materials are available online at https://www.mdpi.com/1660-3397/19/3/130/s1. ESIMS, IR, 1D (^1^H and ^13^C) and 2D (HSQC, HMBC, COSY, and NOESY) NMR spectra of lobocrassin I (**1**) Figures S1–S10 and lobohedleolide (**2**) Figures S10–S19. HRESIMS spectra of lobocrassin I (**1**).
