# Supplementary material for: Cembranoids from Octocoral Lobophytum crassum (von Marenzeller, 1886)
Source: Mar Drugs. 2021 Feb 27;19(3):130. doi: 10.3390/md19030130 (PMC7997245; doi:10.3390/md19030130)
Supplement: Supplementary file 1 [file marinedrugs-19-00130-s001.pdf]

Supporting Information

No	Content	page
Figure S1.	IR spectrum of compound **1**.	2
Figure S2.	ESIMS spectrum of compound **1**.	2
Figure S3.	HRESIMS spectrum of compound **1**.	3
Figure S4.	^{1}H NMR spectrum (400 MHz) of compound **1** in $CDCl_3$.	3
Figure S5.	^{13}C NMR spectrum (100 MHz) of compound **1** in $CDCl_3$.	4
Figure S6.	DEPT spectrum of compound **1** in $CDCl_3$.	4
Figure S7.	HSQC spectrum of compound **1** in $CDCl_3$.	5
Figure S8.	HMBC spectrum of compound **1** in $CDCl_3$.	5
Figure S9.	^{1}H–^{1}H COSY spectrum of compound **1** in $CDCl_3$.	6
Figure S10.	NOESY spectrum of compound **1** in $CDCl_3$.	6
Figure S11.	IR spectrum of compound **2**.	7
Figure S12.	ESIMS spectrum of compound **2**.	7
Figure S13.	^{1}H NMR spectrum (400 MHz) of compound **2** in $CDCl_3$.	8
Figure S14.	^{13}C NMR spectrum (100 MHz) of compound **2** in $CDCl_3$.	8
Figure S15.	DEPT spectrum of compound **2** in $CDCl_3$.	9
Figure S16.	HSQC spectrum of compound **2** in $CDCl_3$.	9
Figure S17.	HMBC spectrum of compound **2** in $CDCl_3$.	10
Figure S18.	^{1}H–^{1}H COSY spectrum of compound **2** in $CDCl_3$.	10
Figure S19.	NOESY spectrum of compound **2** in $CDCl_3$.	11

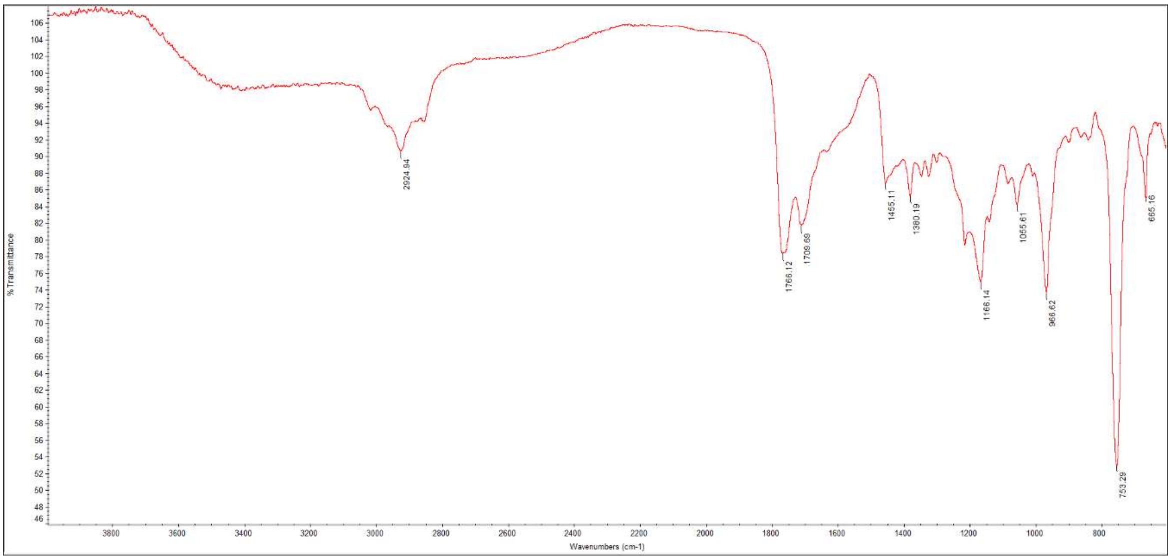

Figure S1. IR spectrum of compound **1**.

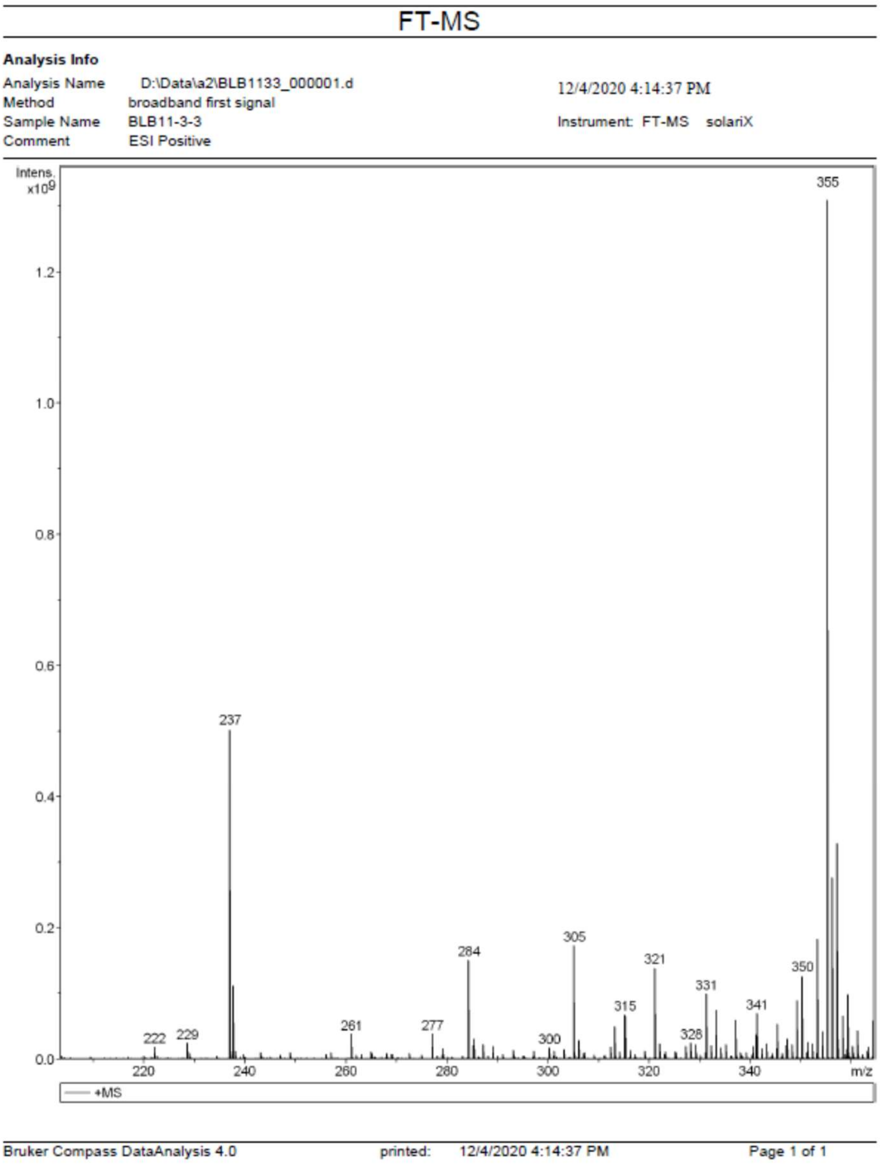

Figure S2. ESIMS spectrum of compound **1**.

Mass Spectrum SmartFormula Report

Analysis Info

Analysis Name	D:\Data\a2\BLB1133_000002.d	12/4/2020 4:13:50 PM
Method	broadband first signal	Operator: YU HSIAO-CHING
Sample Name	BLB11-3-3	Instrument: BRUKER FT-MS solariX
Comment	ESI Positive	

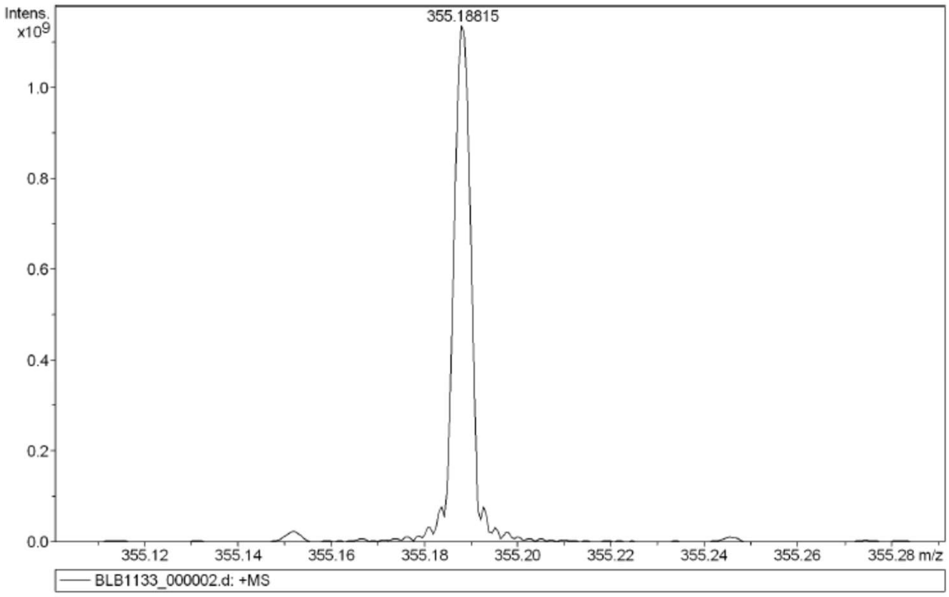

Meas. m/z	#	Formula	Score	m/z	err [mDa]	err [ppm]	mSigma	rdb	e⁻ Conf	N-Rule
355.18815	1	C 20 H 28 Na O 4	100.00	355.18798	-0.17	-0.49	6.1	6.5	even	ok

Figure S3. HRESIMS spectrum of compound **1**.

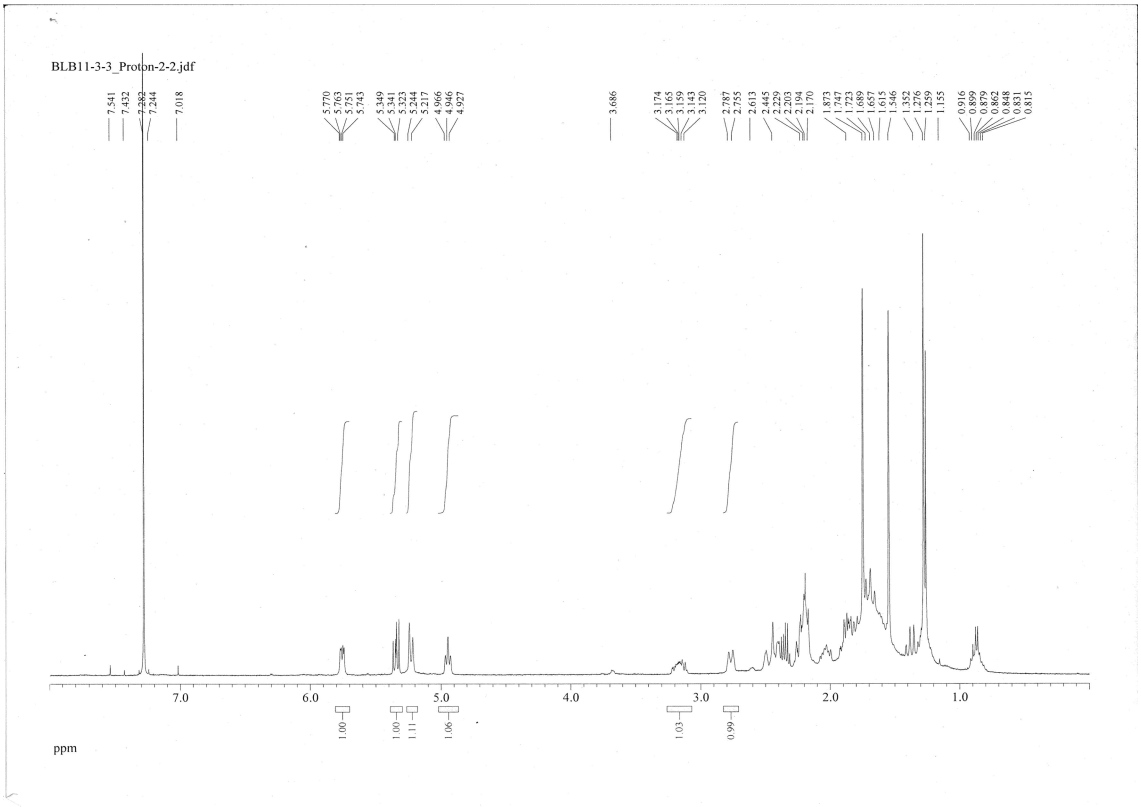

Figure S4. ^{1}H NMR spectrum (400 MHz) of compound **1** in $CDCl_3$.

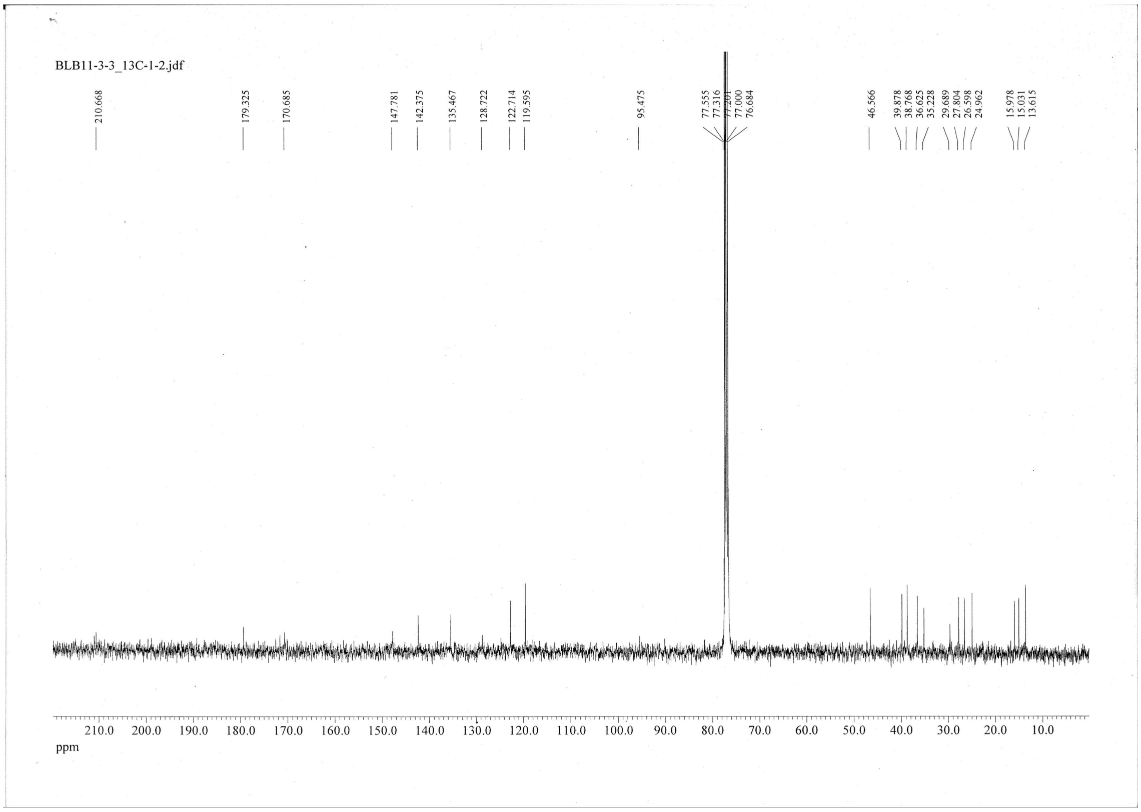

Figure S5. ^{13}C NMR spectrum (100 MHz) of compound **1** in $CDCl_3$.

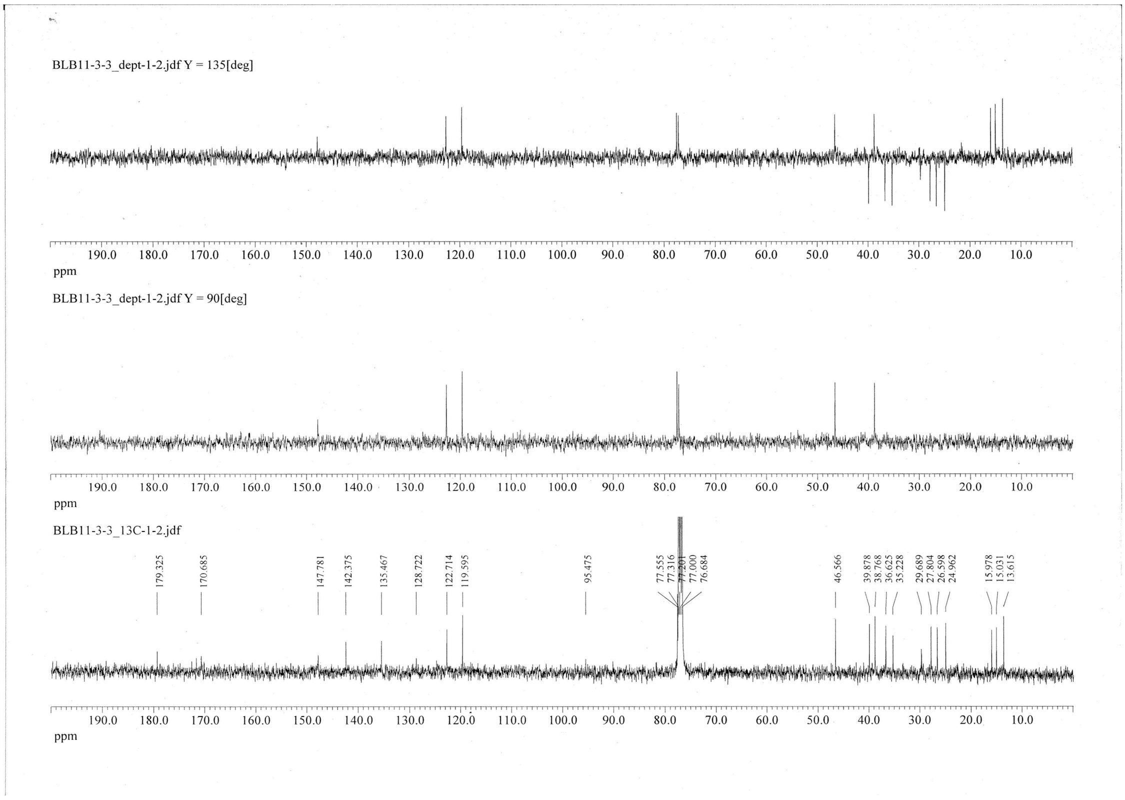

Figure S6. DEPT spectrum of compound **1** in $CDCl_3$.

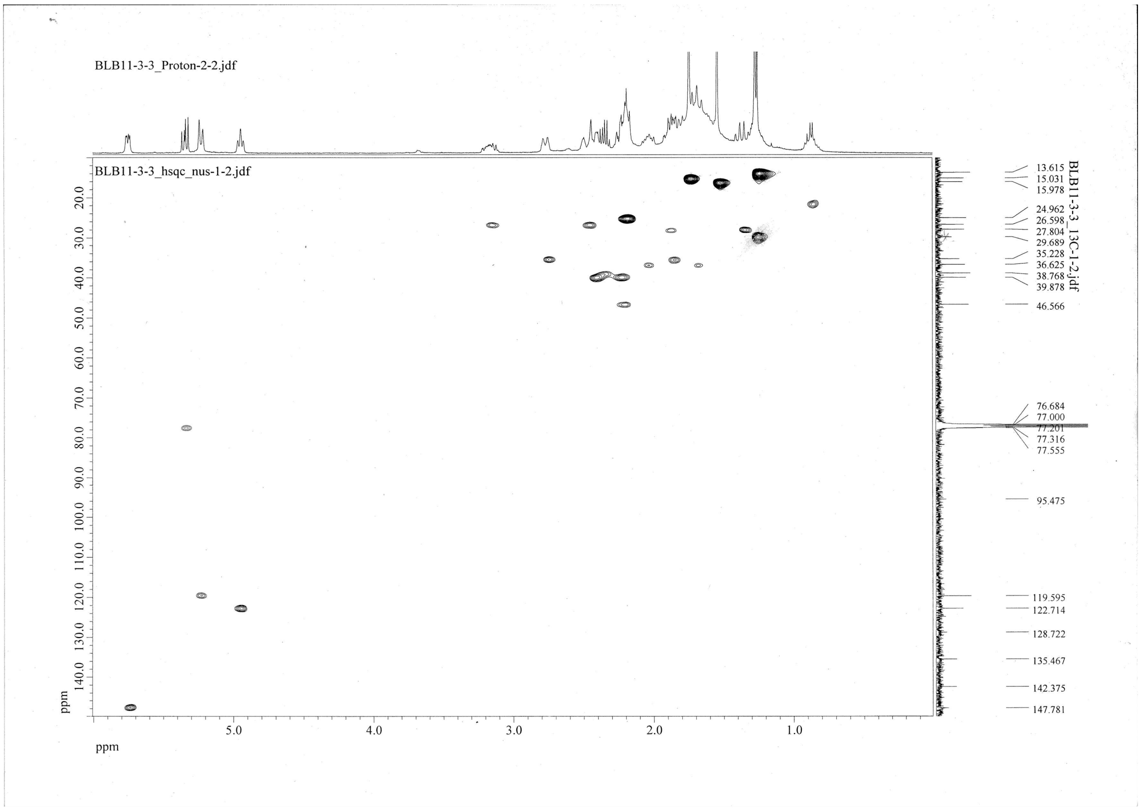

Figure S7. HSQC spectrum of compound **1** in $CDCl_3$.

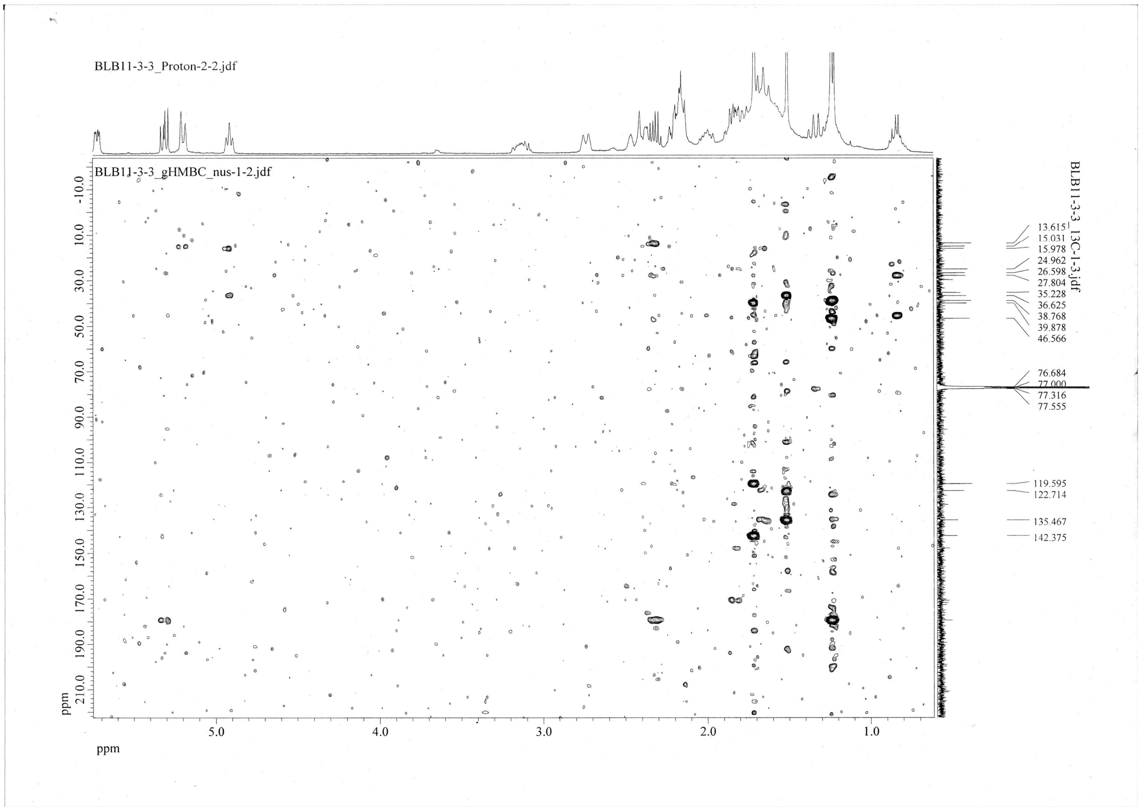

Figure S8. HMBC spectrum of compound **1** in $CDCl_3$.

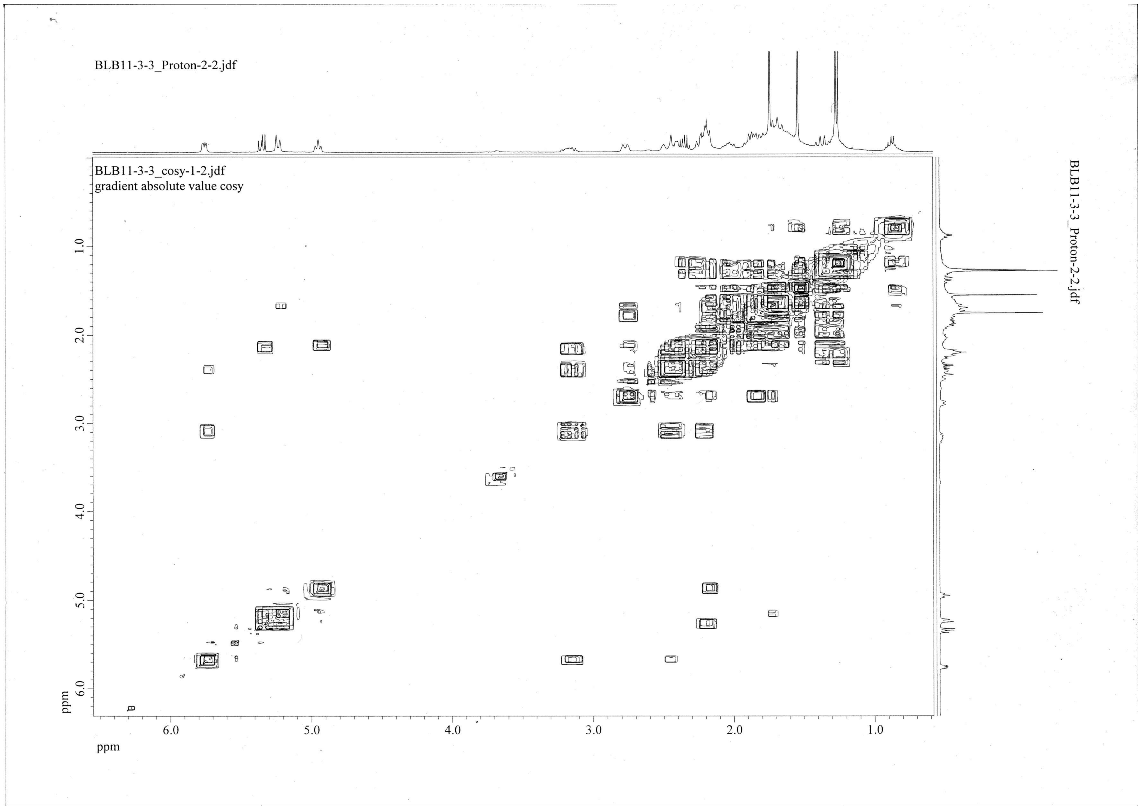

Figure S9. ^{1}H–^{1}H COSY spectrum of compound **1** in $CDCl_3$.

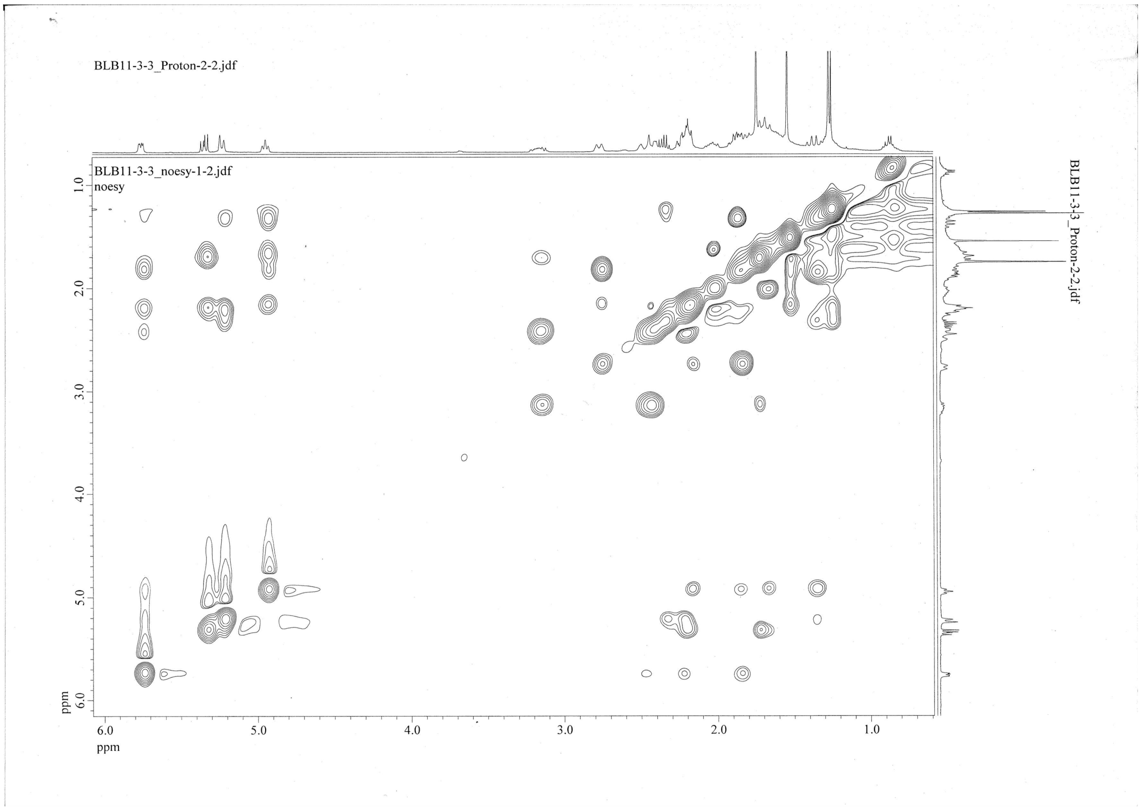

Figure S10. NOESY spectrum of compound **1** in $CDCl_3$.

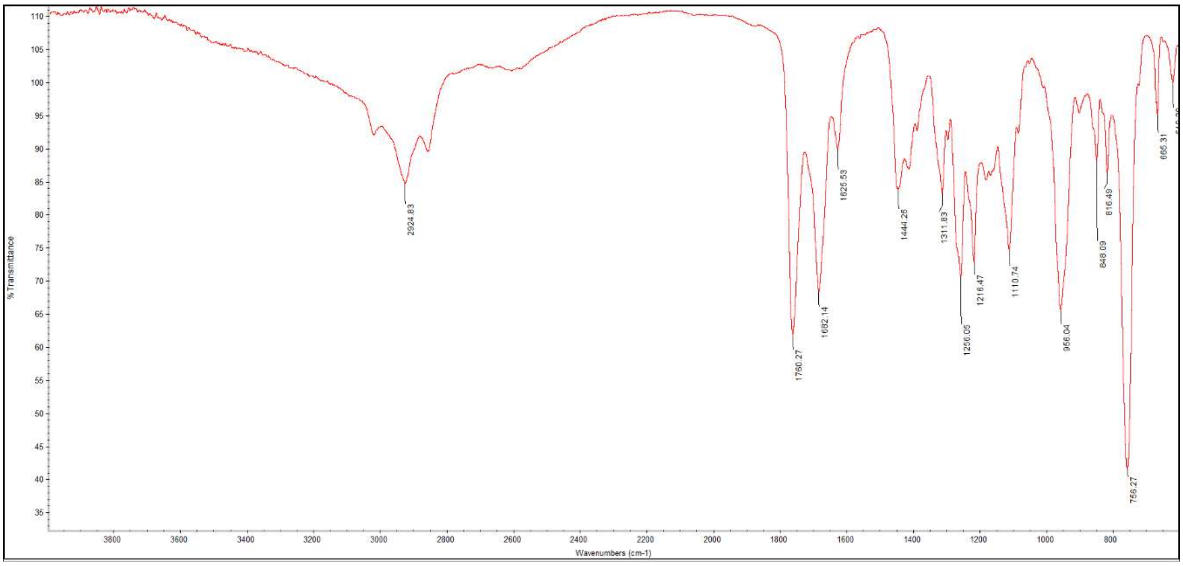

Figure S11. IR spectrum of compound **2**.

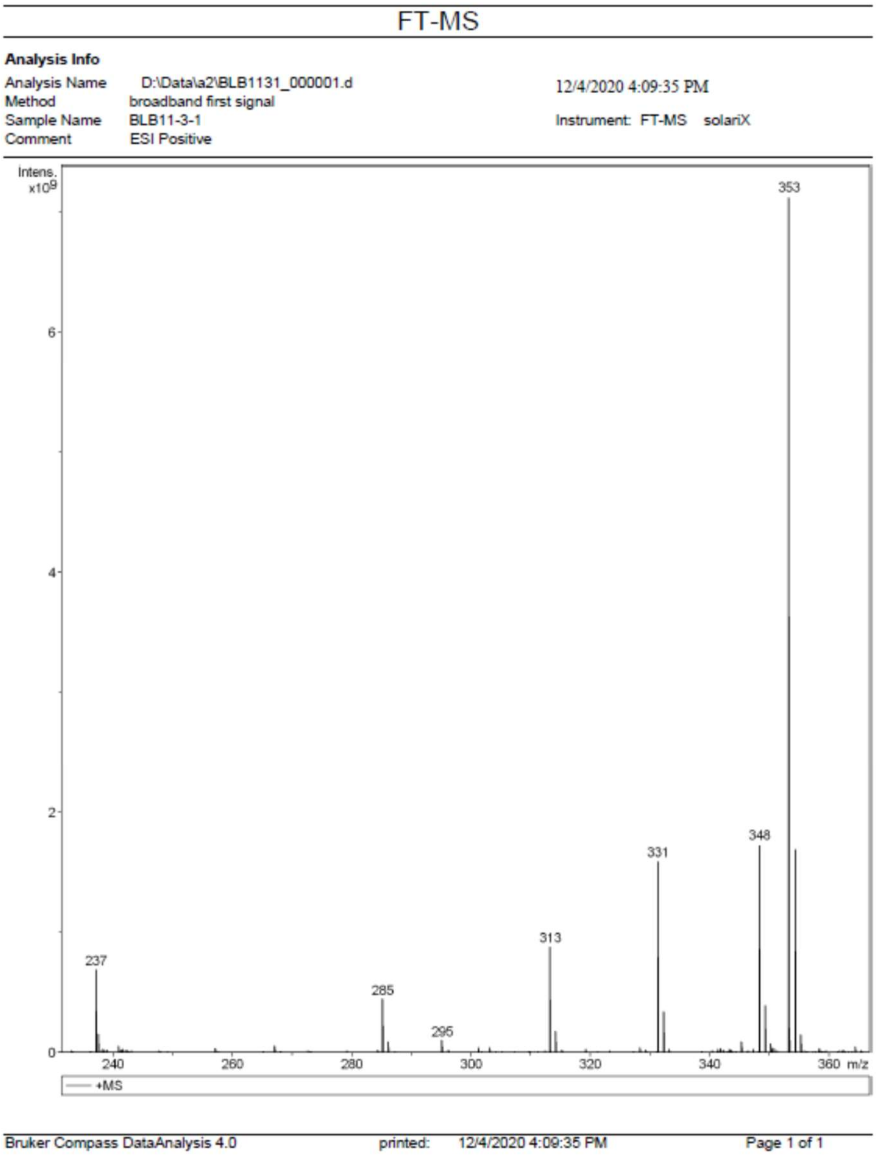

Figure S12. ESIMS spectrum of compound **2**.

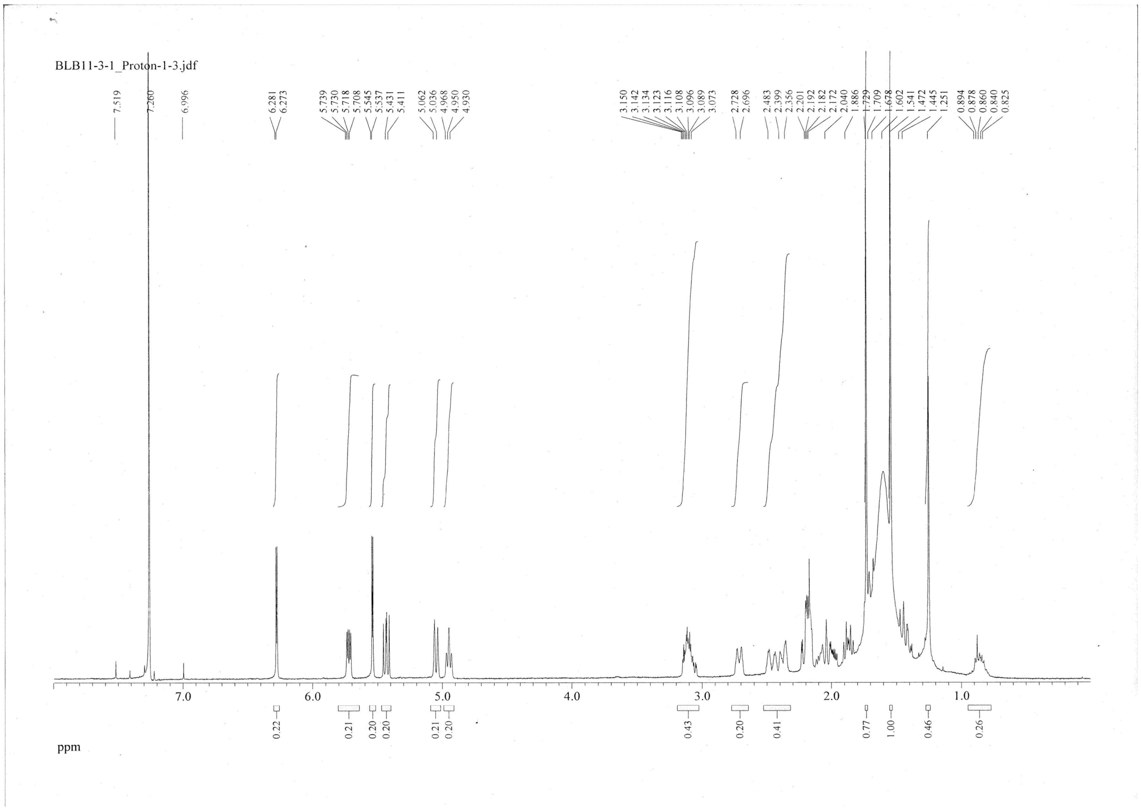

Figure S13. ^{1}H NMR spectrum (400 MHz) of compound **2** in $CDCl_3$.

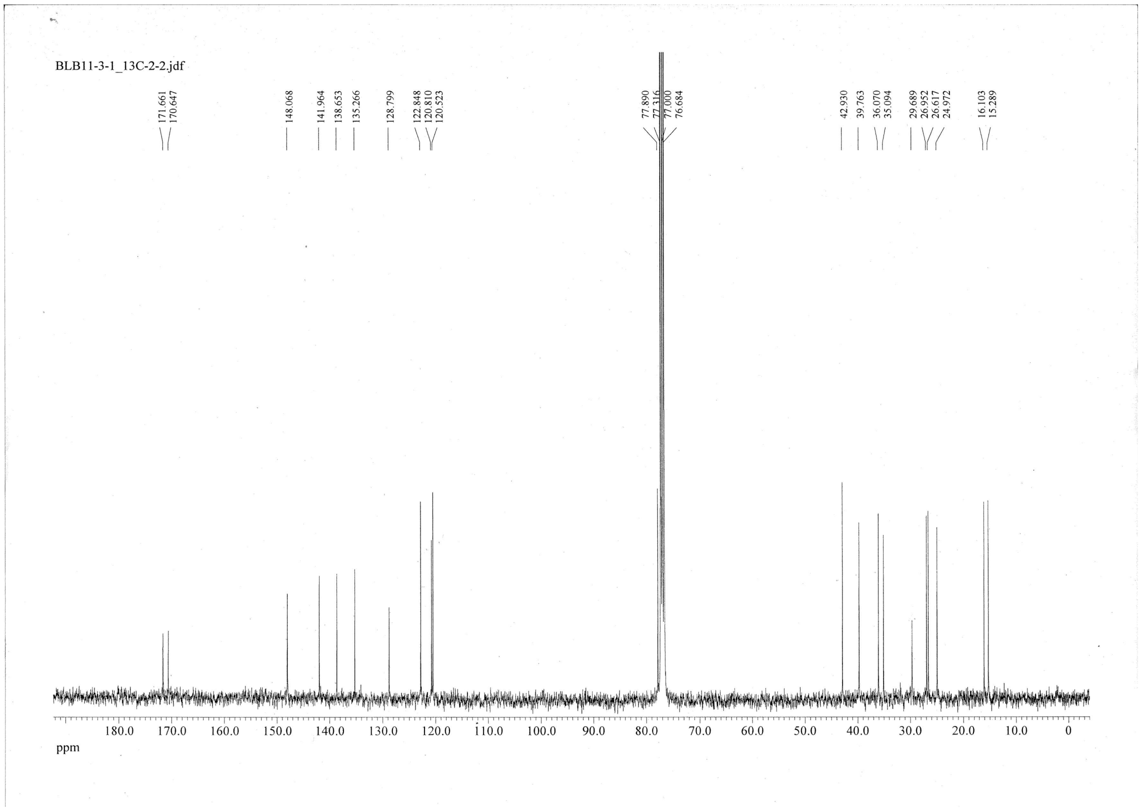

Figure S14. ^{13}C NMR spectrum (100 MHz) of compound **2** in $CDCl_3$.

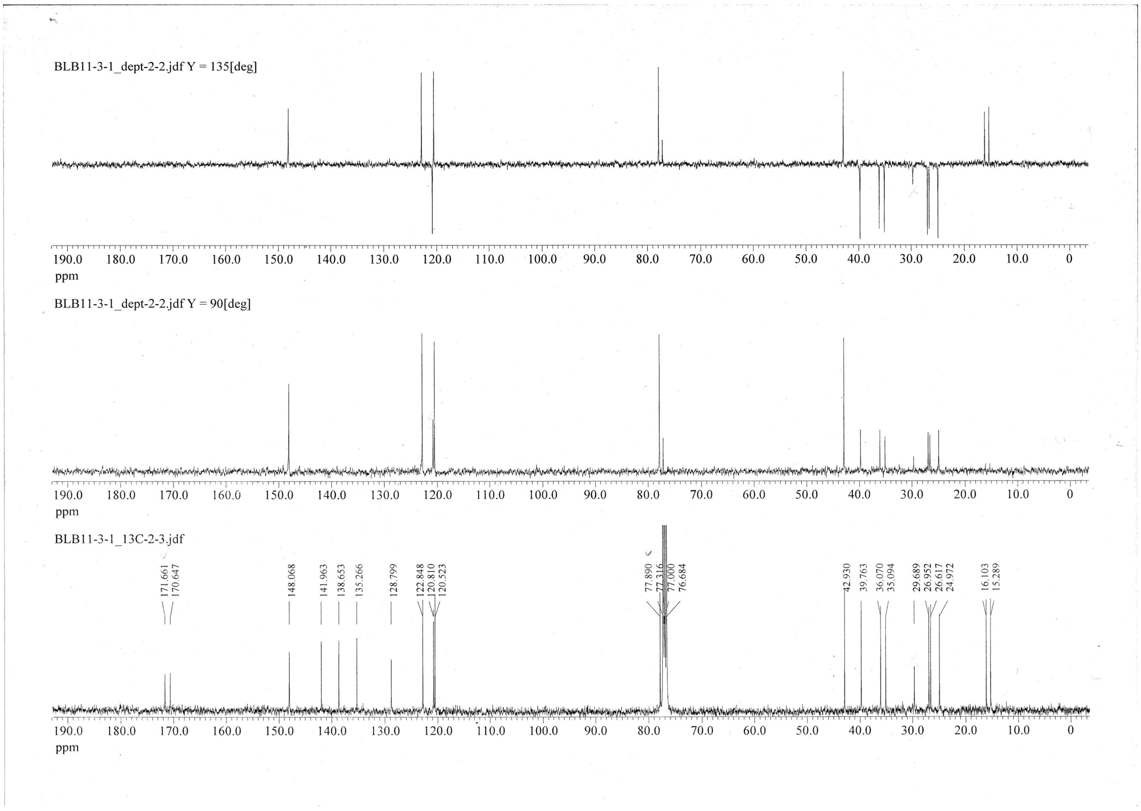

Figure S15. DEPT spectrum of compound **2** in $CDCl_3$.

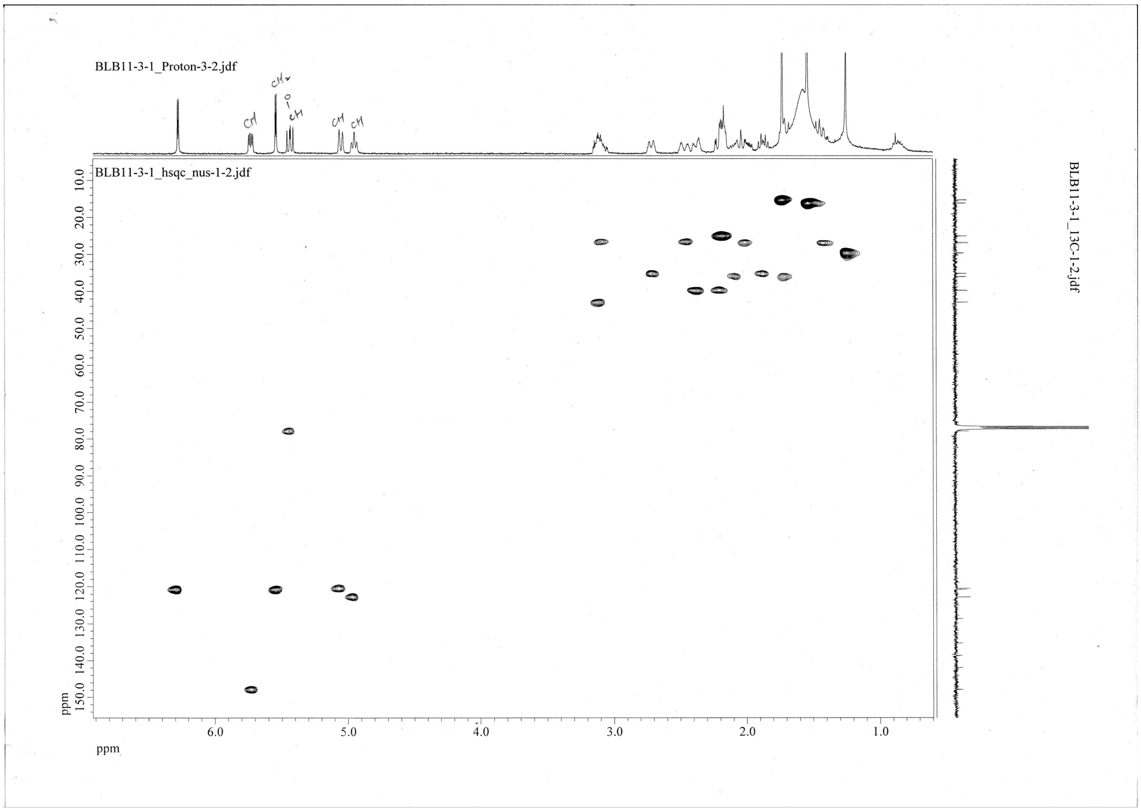

Figure S16. HSQC spectrum of compound **2** in $CDCl_3$.

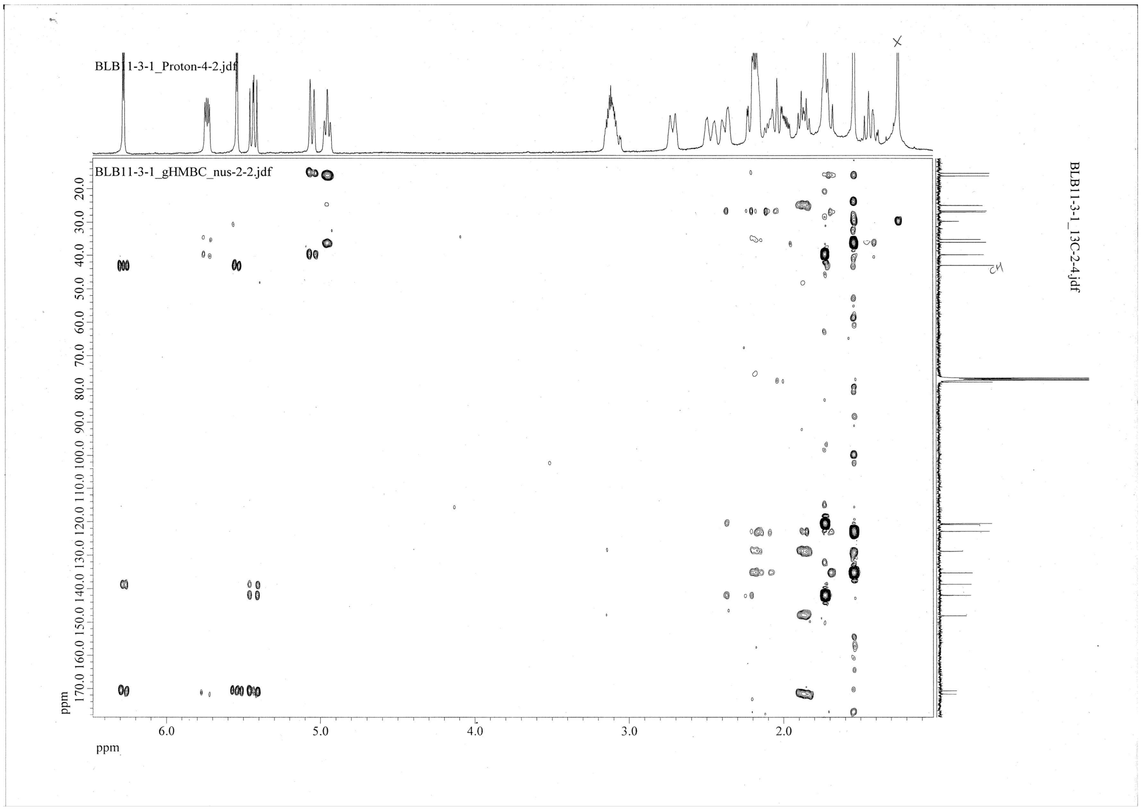

Figure S17. HMBC spectrum of compound **2** in $CDCl_3$.

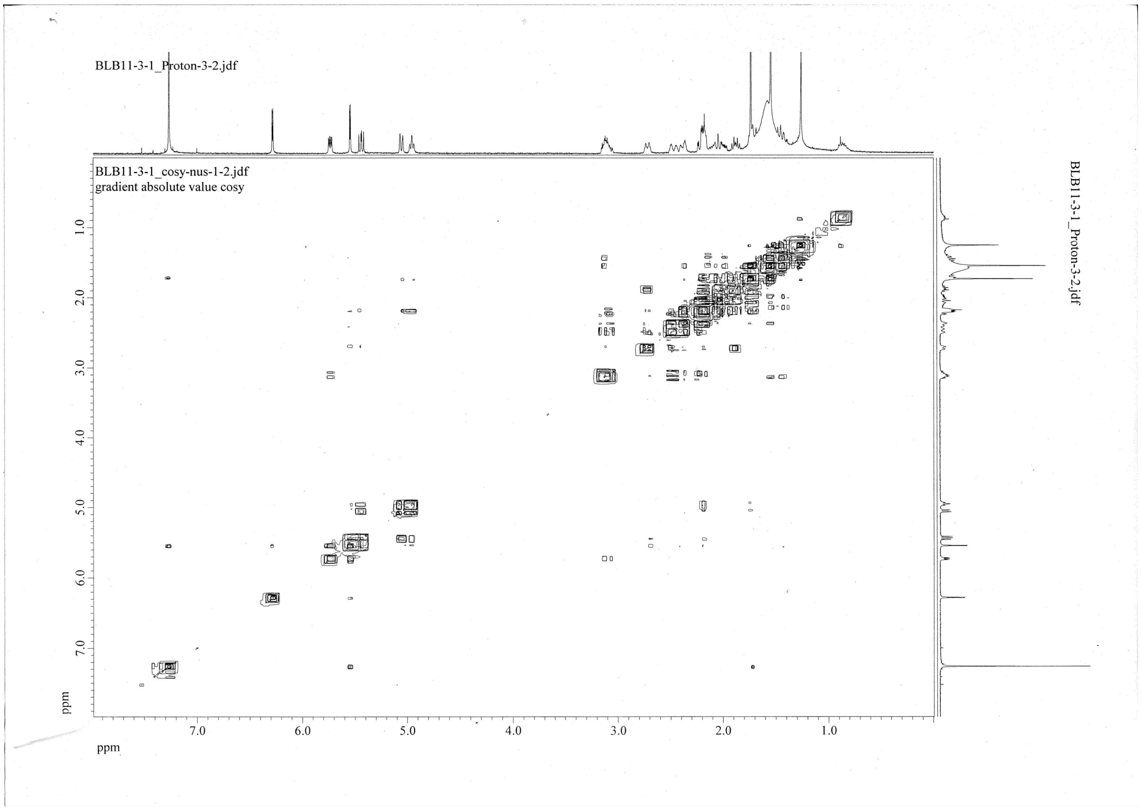

Figure S18. ^{1}H–^{1}H COSY spectrum of compound **2** in $CDCl_3$.

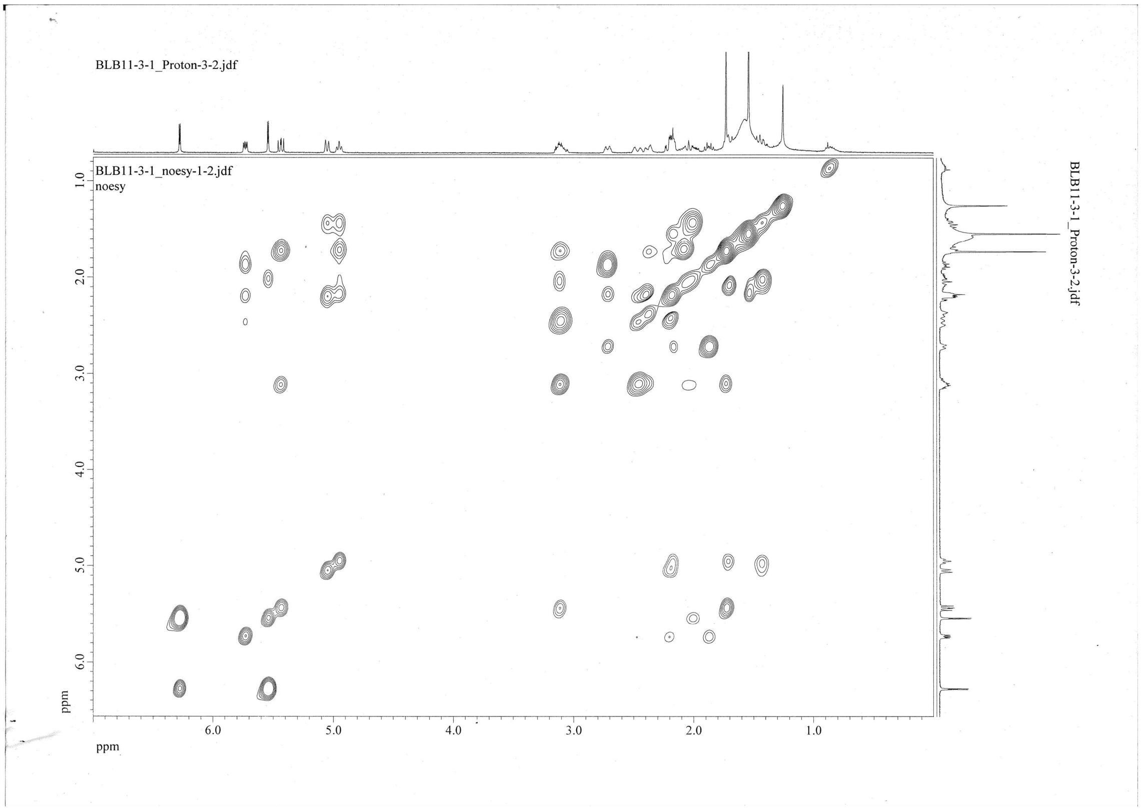

Figure S19. NOESY spectrum of compound **2** in $CDCl_3$.
